# HMGB1 neutralization is associated with bacterial translocation during acetaminophen hepatotoxicity

**DOI:** 10.1186/1471-230X-14-66

**Published:** 2014-04-05

**Authors:** Runkuan Yang, Xiaoping Zou, Jyrki Tenhunen, Shengtao Zhu, Henri Kajander, Marja-Leena Koskinen, Tor Inge Tonnessen

**Affiliations:** 1 Department of Critical Care Medicine, University of Pittsburgh Medical School, 3550 Terrace Street, Pittsburgh, PA 15261, USA; 2 Department of Intensive Care Medicine, Tampere University Hospital, University of Tampere, 10 Bio Katu, Tampere 33014, Finland; 3 Department of Gastroenterology, Drum Tower Hospital, Nanjing University Medical School, 321 Zhongshan Street, 210008 Nanjing, China; 4 Department of Surgical Science, Anesthesiology and Intensive Care Medicine, Uppsala University, 751 85 Uppsala, Sweden; 5 Department of Gastroenterology, Beijing Friendship Hospital, Capital Medical University, 95 Yong An Road, 100050 Beijing, China; 6 Department of Pathology, University of Tampere Medical School, 10 Bio Katu, 33521 Tampere, Finland; 7 Department of Anesthesiology and Intensive Care Medicine, Rikshospital, Oslo University, 4950 Nydalen, 0424 Oslo, Norway

**Keywords:** HMGB1, Acetaminophen, Hepatotoxicity, Gut bacterial translocation

## Abstract

**Background:**

Acetaminophen (APAP) hepatotoxicity is associated with a high rate of gram-negative enteric bacterial infection; however, the underlying mechanism is still unknown. APAP overdose induces massive hepatocyte necrosis, necrotic tissue releases high mobility group B1 (HMGB1) and exogenous HMGB1 is able to induce gut bacterial translocation (BT) in normal mice; therefore, it is possible that HMGB1 mediates gut BT in APAP hepatotoxicity. This study aims to test this hypothesis by using anti-HMGB1 neutralizing antibody to treat APAP overdose for 24-48 hours.

**Methods:**

Male C57BL/6 mice were intraperitoneally (i.p.) injected with a single dose of APAP (350 mg/kg dissolved in 1 mL sterile saline). 2 hrs after APAP injection, the APAP challenged mice were randomized to receive treatment with either anti-HMGB1 antibody (400 μg per dose) or non-immune (sham) IgG every 24 h for a total of 2 doses.

**Results:**

24 and 48 hrs after APAP challenge, anti-HMGB1 treatment instead of sham IgG therapy significantly decreased serum HMGB1 concentrations and reduced BT by 85%; serum HMGB1 levels were positively correlated with the amount of BT; anti-HMGB1 therapy decreased hepatic BT at 48 h, which was associated with better recovered liver structure and better restored hepatic immune system that was shown by enhanced hepatic mRNA expression of TNF-α, IL-6 and extensive proliferation of inflammatory and reticuloendothelial cells; however, anti-HMGB1 treatment did not decrease gut mucosal permeability as compared to the sham IgG therapy at either 24 or 48 hrs.

**Conclusion:**

HMGB1 neutralization is associated with bacterial translocation during APAP hepatotoxicity.

## Background

Acetaminophen hepatotoxicity is the leading cause of drug-induced acute liver failure (ALF) in the United States and other industrialized nations [1]. ALF is associated with a high rate of infectious complications, most of these infections are produced by gram negative enteric bacteria [2]; however, the underlying mechanism is still not clear. APAP overdose induces massive hepatocyte necrosis [3-6] and necrotic tissue passively releases HMGB1 [7-9], a ubiquitous nuclear protein secreted by immunocompetent cells, including monocytes, macrophages and neutrophils, is an important late inflammatory mediator in sepsis [10]. HMGB1 also modulates the inflammatory cascade in activated macrophages [11]. HMGB1 stimulates macrophages to release TNF-α and IL-6, while HMGB1neutralizing antibody can block TNF-α release [11,12] and knocking-out HMGB1 receptor can reverse IL-6 release [12]. HMGB1 contributes to liver injury in ischemia-reperfusion [13] and exogenous HMGB1 injection is able to induce gut hyper-permeability and BT in normal mice [14]; therefore, it is possible that HMGB1 mediates gut bacterial translocation in APAP hepatotoxicity and we tested this hypothesis by using anti-HMGB1 neutralizing antibody to treat APAP overdose for 24 or 48 hours in a murine model.

## Methods

All chemicals were purchased from Sigma-Aldrich Chemical Co. (St. Louis, MO, USA) unless otherwise noted. Polyclonal antibodies against HMGB1 were raised in rabbits (Cocalico Biologicals, Reamstown, PA, USA), and titers were determined by immunoblotting as previously described [15]. Anti–HMGB1 antibodies were affinity-purified by using cyanogen bromide–activated Sepharose beads following standard procedures. Neutralizing activity of anti-HMGB1 was confirmed in HMGB1-stimulated macrophage cultures by assay of TNF-α release. In the presence of anti-HMGB1 antibody, neutralizing antibody was defined as inhibiting > 80% of HMGB1-induced TNF release. Sham IgG antibodies were purified from non-immunized rabbit IgG.

### Ethical considerations

This research protocol complied with the regulations regarding the care and use of experimental animals published by the National Institutes of Health and was approved by the Institutional Animal Use and Care Committee of the University of Pittsburgh Medical School. Male C57Bl/6 mice weighing 20-25 g (Jackson Laboratories, Bar Harbor, ME) were used in this study. The animals were maintained at the University of Pittsburgh Animal Research Center with a 12-hour light-dark cycle and free access to standard laboratory feed and water. Animals were acclimatized for 7 days prior to being studied and fasted overnight prior to the experiments.

### Animal model and experimental groups

In the first experiment, acute liver injury (ALI) was induced by a single dose of APAP (350 mg/kg dissolved in 1 mL sterile saline) administered by i.p. injection. 14 APAP challenged mice were then randomized into the anti-HMGB1 group (n = 6) and the sham IgG group (n = 8). 6 mice injected with saline not containing APAP served as the control group. The animals in the anti-HMGB1 group were given one administration (i.p.injection) of anti-HMGB1 antibody (400 μg dissolved in 0.5 mL sterile saline) 2 hrs after APAP injection. The same amount of sham IgG was i.p. injected to the sham IgG group and the control animals were injected with saline not containing APAP at equivalent time points. 24 hrs after APAP challenge, all surviving mice (2 mice died in the sham IgG group) in each group were anesthetized with sodium pentobarbital (90 mg/kg i.p.).

In the second experiment, ALI was induced the same as above described. APAP injected mice were then randomized into the anti-HMGB1 group (n = 10) and the sham IgG group (n = 11). 6 mice injected with saline not containing APAP served as the control group. The animals in the anti-HMGB1 group were given 2 administrations of anti-HMGB1 antibody (400 μg per dose dissolved in 0.5 mL sterile saline): the first administration was given 2 hrs after APAP injection, the second administration was given 24 hrs after the first administration of anti-HMGB1 antibody. The same amount of sham IgG was given to the sham IgG group and the control animals were injected with saline not containing APAP at equivalent time points. 48 hrs after APAP injection, all surviving mice (2 mice died in the sham IgG group) in each group were anesthetized with sodium pentobarbital (90 mg/kg i.p.).

### APAP bioactivation

The hepatic glutathione concentration was used to estimate APAP bioactivation [16] in sham IgG or anti-HMGB1 treated mice. Frozen liver samples (100 mg) were homogenized in 1 mL of cold buffer containing 0.2 M 2-*N*-morpholino ethansulfonic acid, 50 mM phosphate and 1 mM EDTA [pH 6.0]. Homogenates were centrifuged and supernatants were collected. Total hepatic glutathione concentration in the supernatants was measured using a commercially available kit (Cayman Chemical Co., Ann Arbor, MI).

### Serum HMGB1 concentrations

Blood (1000 μL) was obtained by cardiac puncture; the serum was collected and stored frozen at -80°C. HMGB1 was measured using an HMGB1 ELISA kit purchased from Shino-Test Corporation (Japan, Kanagawa 229-0011).

### Serum aminotransferase measurements

Blood was obtained by cardiac puncture and serum levels of ALT were measured at 37°C with a commercially available kit (Sigma Diagnostic).

### Intestinal mucosal permeability

Ileal mucosal permeability to the fluorescent tracer, FITC-dextran with a molecular mass of 4000 Da (FD4), was determined using an everted gut sac method, as previously described [14,15,17]. In brief, everted gut sac were prepared in ice-cold modified Krebs-Henseleit Bicarbonate Buffer (KHBB) (pH = 7.4). One end of the gut segment was everted onto a thin plastic rod. The resulting sac was secured with another suture to the grooved tip of a 3-mL plastic syringe containing KHBB. We gently distended the sac by injecting 1.5 mL of KHBB. The sac was then suspended for 30 minutes in a 50 mL beaker containing 40 mL of KHBB plus FD-4 (40 mg/mL). The solution in the beaker was temperature-jacketed at 37°C and continuously bubbled with a gas mixture containing 95% O_2_-5% CO_2_. The FD4 concentration of the fluid in the beaker and inside of the sac was determined spectroflurometrically, and permeability was expressed as the mucosal-to-serosal clearance of FD-4.

### Estimation of bacterial translocation

ALF patients are prone to infection, sepsis and multiple organ failure (MOF) [2]. In order to test the hypothesis that HMGB1 mediates BT in APAP hepatotoxicity, BT to the mesenteric lymph nodes (MLN) and to the liver was determined as previously described [14,15,17]. Briefly, the skin was cleaned with 10% povi-done-iodine. Using sterile technique, the abdominal cavity was opened and the viscera were exposed. The MLN complex and a piece of liver tissue from the left lobe were removed, weighed, and placed in a grinding tube containing 0.5 mL ice-cold PBS. The MLN and liver tissue were homogenized with glass grinders, and a 250-μL aliquot of the homogenate was plated onto brain-heart infusion and MacConkey’s agar (Becton Dickinson, Sparks, MD, USA). The plates were examined 24 h later after being aerobically incubated at 37°C. The colonies were counted and the results were expressed as colony-forming units (CFU) per gram of tissue.

### Gut histopathology

A segment of terminal ileum tissue was fixed in 10% formalin buffer. The paraffin-embedded ileum was sectioned, stained with hematoxylin and eosin (H&E) and examined using light microscopy. Blind analysis was performed on all samples to determine the degree of lesion per microscopic field at a magnification of 200×. Five fields were evaluated per tissue sample. Histological injury was graded by pathologist using a standardized injury scoring system according to Zhang et al. [18] with modifications as follows: Grade 0, no injury; Grade 1, epithelial cells of the villi became swollen, cell structure especially nuclei became unclear; Grade 2, loss of mucosal epithelial lining of the villous tip; Grade 3, loss of mucosal epithelial lining in area more than the villous tip.

Grade 4, less than 10% of the villi structure became fragile to break; Grade 5, more than 30% of the villi became fragile to break.

### Real time RT-PCR

Hepatic mRNA levels of pro-regeneration cytokines TNF-α, IL-6 were determined by real time RT-PCR as described [19]. Total RNA was isolated with TRIzol reagent according to the manufacturer’s instructions (Invitrogen, Carlsbad, CA). To determine the relative amount of cytokine messenger RNA, amplification of sample complementary DNA was monitored using the Smart Cycler (Cepheid, Sunnyvale, CA) and the DNA fluorescent dye SYBR Green (Molecular Probes, Eugene, OR). Primers were designed using reported sequences in GenBank. Primer sequences are as follows: interleukin (IL)-6: 5′GAGGATACCACTCCCAACAGACC, 3′ AAGTGCATCATCGTTGTTCATACA; tumor necrosis factor (TNF-α): 5′ GGGACAGTGACCTGGACTGT, 3′ CTCCCTTTGCAGAACTCAGG; glyceraldehyde-3-phosphate dehydrogenase: 5′ GTCTTCACCACCATGGAGAAG, 3′ CCACCTTCTTGATGTCATCAT. Primers were designed to span at least one intron, if possible, to minimize risk of genomic amplification. The specificity of the primers was verified via analysis of polymerase chain reaction (PCR) product with ethidium bromide-stained agarose gel electrophoresis as well as direct sequencing of the PCR product. All real-time PCR runs included a melting curve analysis to assure the specificity of the product with each PCR reaction.

### Proliferating cell nuclear antigen (PCNA) staining

The liver plays an important role in the process of BT [2] by virtue of its many phagocytic cells comprising about 80% to 90% of the reticuloendothelial system function in the body [20]. Therefore, the PCNA imunohistochemistry was carried out to confirm liver regeneration, especially the proliferation of the non-parenchyma cells, such as Kupffer cells, NK and other immune cells in the liver, because these cells exert cellular defense functions for the whole body but also for the liver itself and these cells might also be severely damaged in the APAP hepatotoxicity. Therefore, sections from mouse liver following APAP challenge were prepared and processed for immunohistochemistry using PCNA staining kits from Invitrogen (Camarillo, CA, USA) according to the manufacturer’s instructions. In brief: A piece of liver tissue from the left lobe was fixed in 10% formalin buffer. Paraffin-embedded liver sections were dewaxed by treating the slides in 2 changes of xylene for 5 minutes each and were rehydrated. Endogenous peroxidase activity was quenched by using 0.3% H_2_O_2_ in PBS. The slides were incubated with antigen retrieval solution at 89°C for 10 minutes, then allow the solution to slowly cool down to room temperature for 20 minutes. The slides were incubated with ready-to-use blocking solution for 10 minutes and then incubated with biotinylated monoclonal anti-PCNA antibody (ready-to-use) for 1 hour. After washing, sections were incubated with Streptavidin-Peroxidase (ready-to-use) for 10 minutes at room temperature, followed by incubation with 3, 3′-diaminobenzidine (DAB) for 5 minutes. Slides were finally counterstained with hematoxylin. Digital images of 5 high-power fields from each liver sample were obtained in a random and blinded fashion, and the number of PCNA-labeled nuclei was counted. The average number of PCNA-positive cells in each animal was used for subsequent analysis.

### Hepatocyte proliferation by 5-bromo-2-deoxyuridine (BrdU) staining

We have found that the previous Invitrogen PCNA kits stain non-parenchymal cells better than hepatocytes [21], and the BrdU kits stain hepatocytes better than the inflammatory cells in APAP overdose [22], suggesting that the BrdU kits are more specific and suitable for hepatocytes staining, and Invitrogen PCNA kits are more specific for staining hepatic non-parenchymal cells, this notion is further supported by the fact that on the standard positive control slide provided by the PCNA kits, a large number of immune/inflammatory cells in the gut lamina propria are also stained PCNA positive. Therefore, we compared the two different staining kits in the same experiment. Since at 24 h time point, the APAP challenged C57/BL6 mice only showed occasional hepatocyte nuclei labeled for 5-bromo-2-deoxyuridine (BrdU) [23], BrdU test was performed only at 48 h time point in this study. To evaluate hepatocyte regeneration, mice from the 48 h groups were administered BrdU (50-mg/kg i.p. injection at 46 h time point) 2 hours before they were killed. A piece of liver tissue from the left lobe was fixed in 10% formalin buffer. Paraffin-embedded liver sections were prepared and processed for immunohistochemistry using BrdU *in-situ* staining kits from BD Pharmingen (San Jose, CA, USA) according to the manufacturer’s instructions. In brief: Paraffin-embedded liver sections were deparaffinized by treating the slides in 2 changes of xylene for 5 minutes each and were rehydrated. Endogenous peroxidase activity was quenched by using 0.3% H_2_O_2_ in PBS. The slides were incubated with antigen retrieval solution at 89°C for 10 minutes, then allow the solution to slowly cool down to room temperature for 20 minutes. The slides were incubated with an anti-BrdU antibody (1:10) for 1 hour. After washing, sections were incubated with ready-to-use Streptavidin-HRP for 30 minutes at room temperature, followed by incubation with 3, 3′-diaminobenzidine (DAB) for 5 minutes. Slides were finally counterstained with hematoxylin. Digital images of 5 high-power fields from each liver sample were obtained in a random and blinded fashion, and the number of BrdU-labeled hepatocyte nuclei was counted. The average number of BrdU-positive hepatocytes in each animal was used for subsequent analysis.

### Statistical methods

Data are presented as means ± SEM. Bacterial translocation data CFU between groups were analyzed using Wilcoxon U-test. Data are presented as means ± SEM. Continuous data were analyzed using two-tailed student’s t-test or analysis of variance followed by Fisher’s LSD test. The statistical analysis was performed by GraphPad Prism 5.0 (La Jolla). Significance was accepted at the 5% level.

## Results

### APAP bioactivation

The hepatic glutathione concentration was used to estimate APAP bioactivation in sham IgG or anti-HMGB1 treated mice. Mice treated only with saline had 5.9 ± 0.4 μmol of glutathione/g liver tissue. 6 hours after APAP injection (sham IgG or anti-HMGB1 antibody was injected 2 hours after APAP challenge), glutathione concentrations were 0.69 ± 0.066 and 0.73 ± 0.08 μmol of glutathione/g liver in mice treated with sham IgG and anti-HMGB1 neutralizing antibody, respectively. There was no significant difference between the sham IgG group and the anti-HMGB1 therapy group (n = 5 for each group).

### Serum HMGB1 concentrations

**Figure 1:**
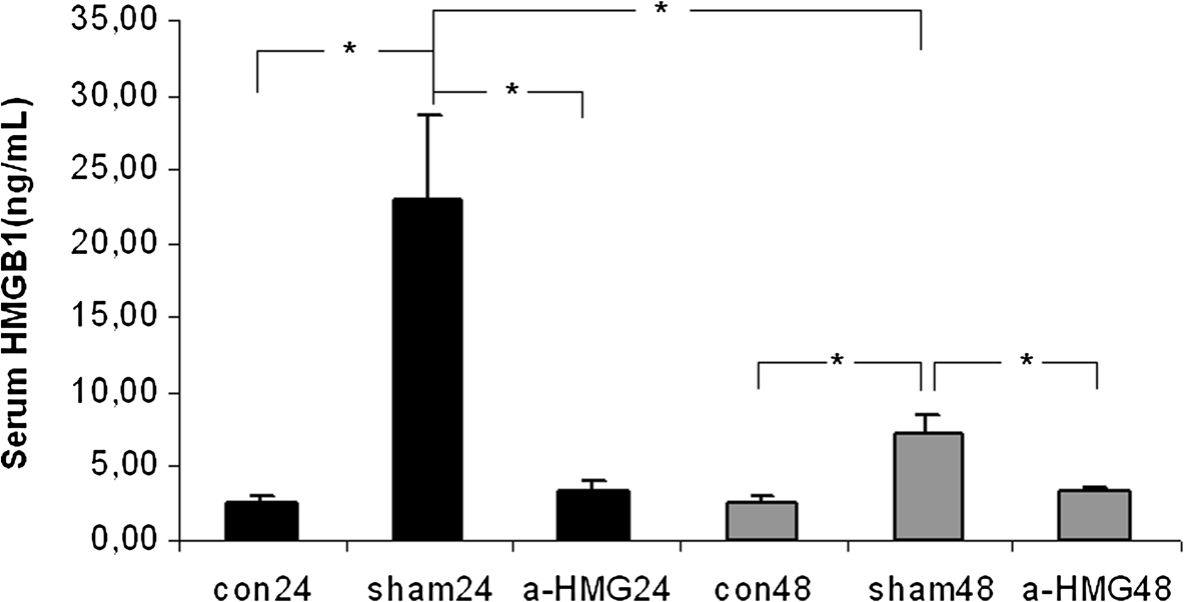
**Effect of treatment with anti-HMGB1 antibody or sham IgG on serum HMGB1 concentrations in acetaminophen (APAP)-induced acute liver injury (ALI).** ALI was induced in C57 BL/6 male mice with a single dose of APAP (350 mg/kg dissolved in 1 mL saline) by intraperitoneal (i.p.) injection. 2 hrs after the APAP injection, 400 μg of anti-HMGB1 antibody in 0.5 mL saline was i.p. injected into mice in the anti-HMGB1 group, the same amount of sham IgG or saline alone was given to the sham IgG group and the control animals at the equivalent time points. The same amount of anti-HMGB1 or the sham IgG was repeated 24 hours after the first administration. Serum HMGB1 concentrations were measured 24 hours after APAP injection from the anti-HMGB1 group, the sham IgG group and the control group (n = 6 for each group). 3 separate groups of mice were used to measure serum HMGB1 concentrations at 48 h time point from the anti-HMGB1 group (n = 10), the sham IgG group (n = 9) and the control group (n = 6). Results are means ± SEM. *indicates p < 0.05 (analysis of variance followed by Fisher’s LSD test).

24 hours and 48 hours after APAP challenge, serum HMGB1 concentrations in the sham IgG groups and the anti-HMGB1 groups were significantly increased as compared to the control groups; the 24 h serum HMGB1 level in the sham IgG group was significantly higher than the 48 h serum HMGB1 level in the sham IgG group; 24 h after APAP injection, serum HMGB1 concentration in the anti-HMGB1 group was significantly lower than that in the sham IgG groups, and at 48 h time point, serum HMGB1 concentration in the anti-HMGB1 group was statistically lower than that in the sham IgG group (Figure 1).

### Bacterial translocation to MLN and the liver

**Figure 2:**
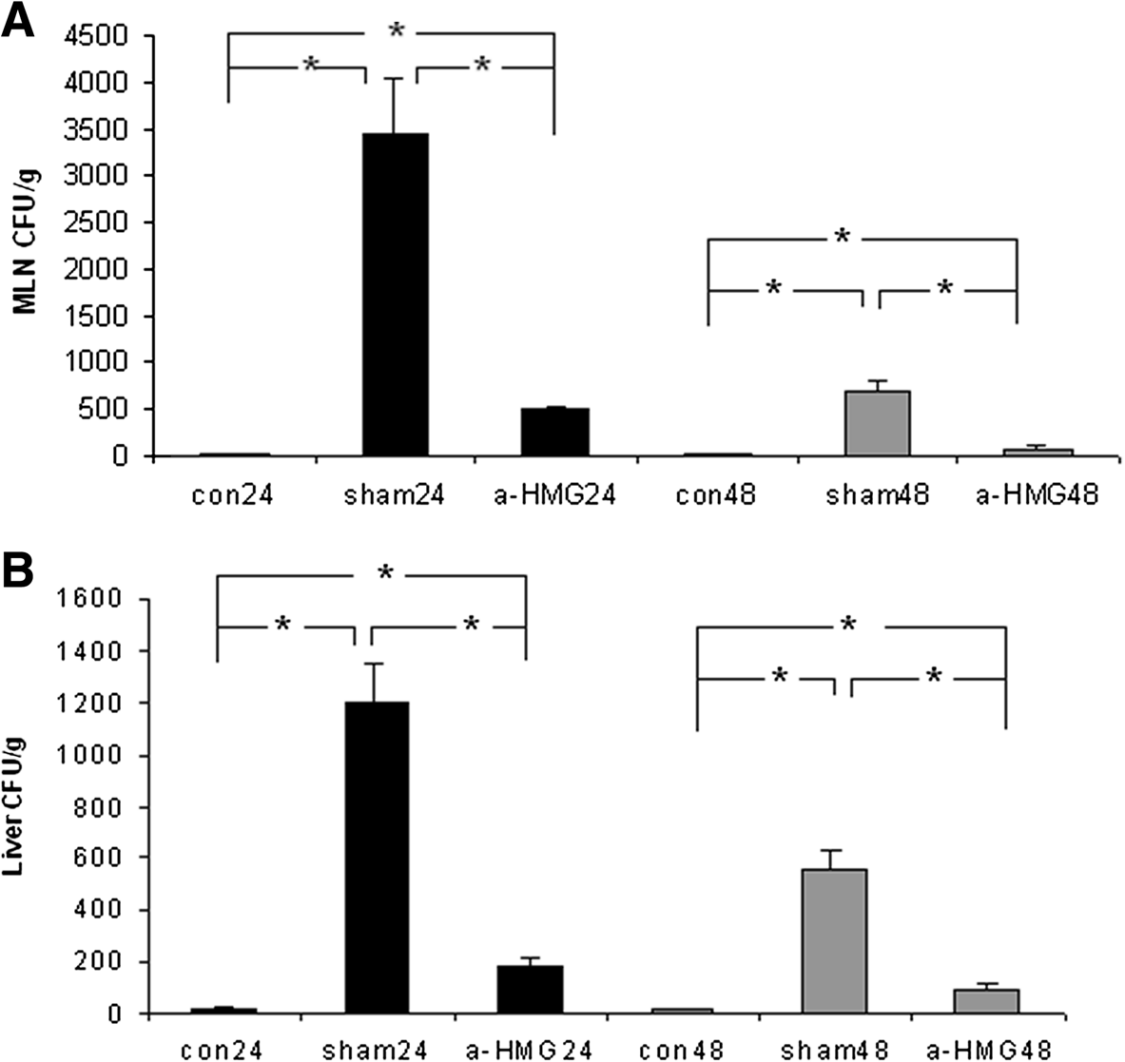
**Effect of treatment with anti-HMGB1 antibody or sham IgG on bacterial translocation in mice with acetaminophen overdose. A**: Bacterial translocation (BT) to mesenteric lymph nodes (MLN) was assessed 24 and 48 hrs after APAP injection. **B**: Bacterial translocation to the liver was assessed 24 and 48 hrs after APAP injection. The animal model and treatment were the same as described in Figure 1. Results are means ± SEM. *indicates p < 0.05 (Wilcoxon U-test).

The extent of bacterial translocation was measured in our published APAP toxicity studies [21,24] (BT data were not published) and the results showed that: when i.p. injected into mice, APAP (300 mg/kg in 1 ml saline) promoted BT as evidenced by greater numbers of viable colony-forming units (CFU) compared with control mice (injected with saline alone) when MLN homogenates were cultured. The number of CFU (in the APAP injected mice) began to increase at 6 h, reached the peak at 24 h, and then the number of CFU decreased at 48 h, by 72 hours, the bacterial translocation was not statistically greater than that in the controls animals. Based on above information, 24 h and 48 h time points were selected to study BT in this investigation. Our study showed that bacterial translocation (to MLN and the liver) was minimal in the control groups, however, there was extensive bacterial translocation (to MLN and the liver) in the sham IgG groups at both 24 h and 48 h time points, and about 85% of BT in APAP injected mice could be reversed by anti-HMGB1 treatment at both 24 h and 48 h (Figure 2). In addition, the amount of BT was positively correlated with serum HMGB1 levels (*R*^*2*^ *= 0.95*).

### Gut mucosal permeability in APAP challenged mice

**Figure 3:**
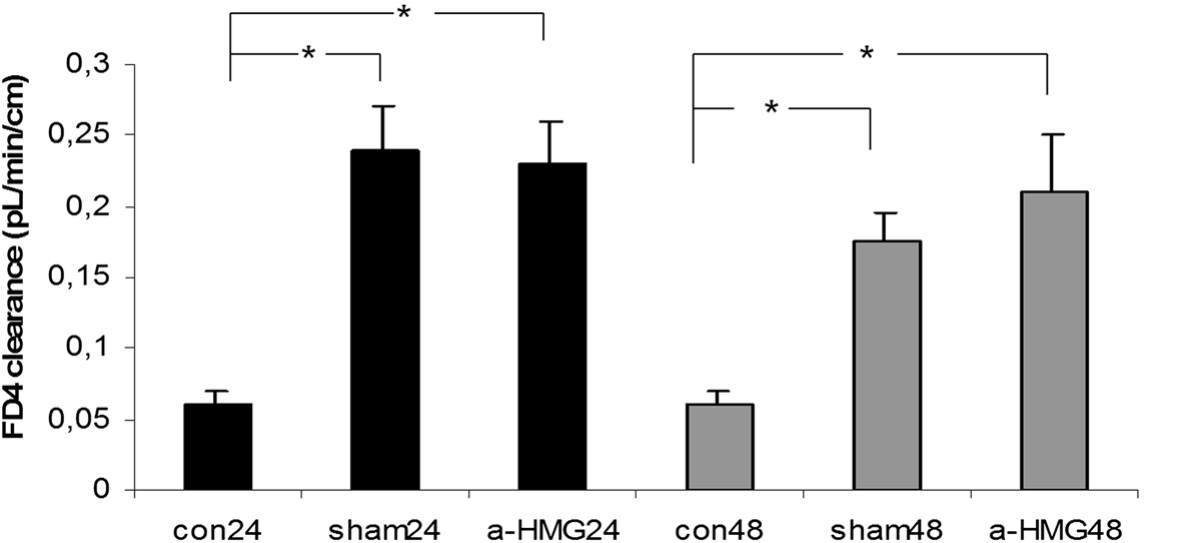
**Effect of treatment with anti-HMGB1 antibody or sham IgG on gut mucosal permeability in mice with acetaminophen overdose.** Ileal mucosal permeability was assessed 24 and 48 hrs after acetaminophen administration. The animal model and treatment were the same as described in Figure 1. Results are means ± SEM. *indicates p < 0.05 (student’s T test).

Compared to the control group, ileal mucosal permeability was significantly increased in APAP challenged mice at both 24 h and 48 h time points; compared to the sham IgG treatment, the anti-HMGB1 therapy did not reduce mucosal permeability at either 24 h or 48 h (Figure 3).

### Gut mucosal injury in APAP challenged mice

**Figure 4:**
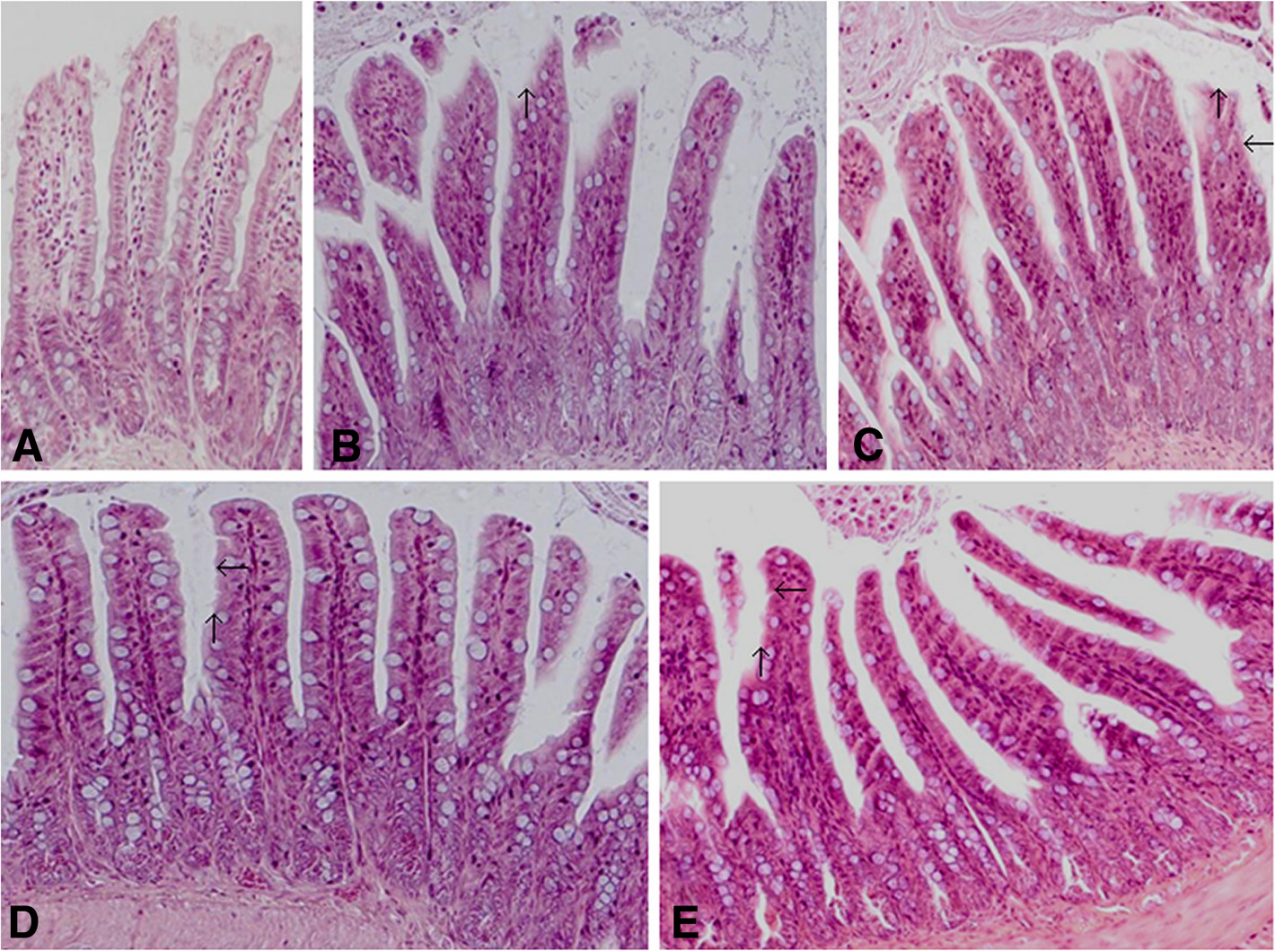
**Effect of treatment with anti-HMGB1 antibody or sham IgG on gut mucosal injury in mice with acetaminophen overdose.** Hematoxylin-eosin staining of the terminal ileum was assessed 24 and 48 hours after acetaminophen administration. Animal model and treatment were the same as described in Figure 1 (n = 6 for each group at 24 h; at 48 h time point, n = 9 for the sham IgG group and n = 10 for the anti-HMGB1 group. **A** = control, **B** = sham IgG at 24 h, **C** = anti-HMGB1 at 24 h, **D** = sham IgG at 48 h, **E** = anti-HMGB1 at 48 h, magnification = 200× for each photo). Typical picture is shown. Arrow indicates the loss of mucosal epithelial line of the villi.

All mice subjected to APAP injection had significantly increased histological injury scores at both 24 h and 48 h time points as compared to the control animals (n = 6, injury score = 0, *p < 0.05*). 24 h after APAP injection, the injury score in the sham IgG group (4.3 ± 0.6, n = 6) was not significantly different from the score in the anti-HMGB1 group (4.1 ± 0.7, n = 6); at 48 h, the injury score in the anti-HMGB1 therapy group (3.3 ± 0.5, n = 10) was not significantly lower than that in the sham IgG group (3.6 ± 0.4, n = 9). Results are means ± SEM (Figure 4).

### Hepatic mRNA expression of TNF-α, IL-6

**Figure 5:**
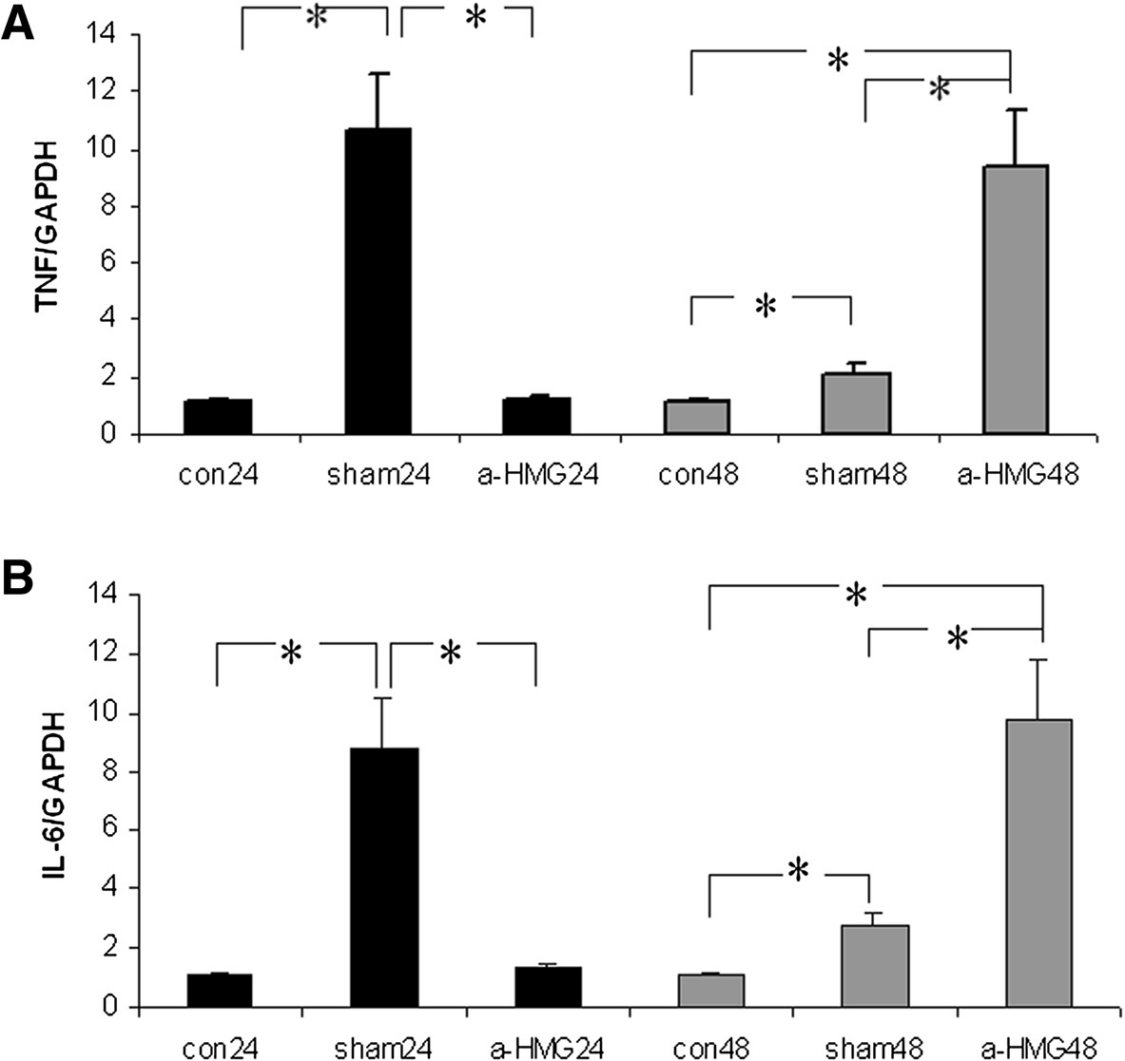
**Effect of treatment with anti-HMGB1 or sham IgG on the mRNA expression of hepatic TNF-α and IL-6 in mice challenged with acetaminophen overdose.** 24 and 48 hours after APAP administration, relative messenger RNA levels of **(A)** TNF-α and **(B)** IL-6 were detected using real-time PCR. Animal model and treatment were the same as described in Figure 1. Baseline (control) levels of cytokines were measured 24 and 48 hrs after saline injection. *indicates p < 0.05 (analysis of variance followed by Fisher’s LSD test). GAPDH = glyceraldehydes-3-phosp dehydrogenase.

Real time RT-PCR was used to detect mRNA levels of TNF-α, IL-6 in hepatic tissue, all of which have previously been implicated in priming hepatocyte regeneration of toxin-induced liver injury [25]. 24 hours after APAP injection, compared to the control group, mRNA levels of TNF-α and IL-6 were markedly increased in the sham IgG treatment group (p *< 0.05*); blockade of HMGB1 significantly decreased hepatic mRNA levels of TNF-α, IL-6 as compared to the sham IgG treatment (*p < 0.05*); 48 hours after APAP administration, compared to the control group, sham IgG treatment statistically increased TNF-α and IL-6 mRNA levels (*p < 0.05*); anti-HMGB1 therapy significantly increased hepatic TNF-α, IL-6 mRNA levels as compared to the sham IgG treatment (*p < 0.05*). Baseline levels of all the cytokines remained low after saline injection in the control group (Figure 5).

### Hepatic proliferating cell nuclear antigen (PCNA) staining

**Figure 6:**
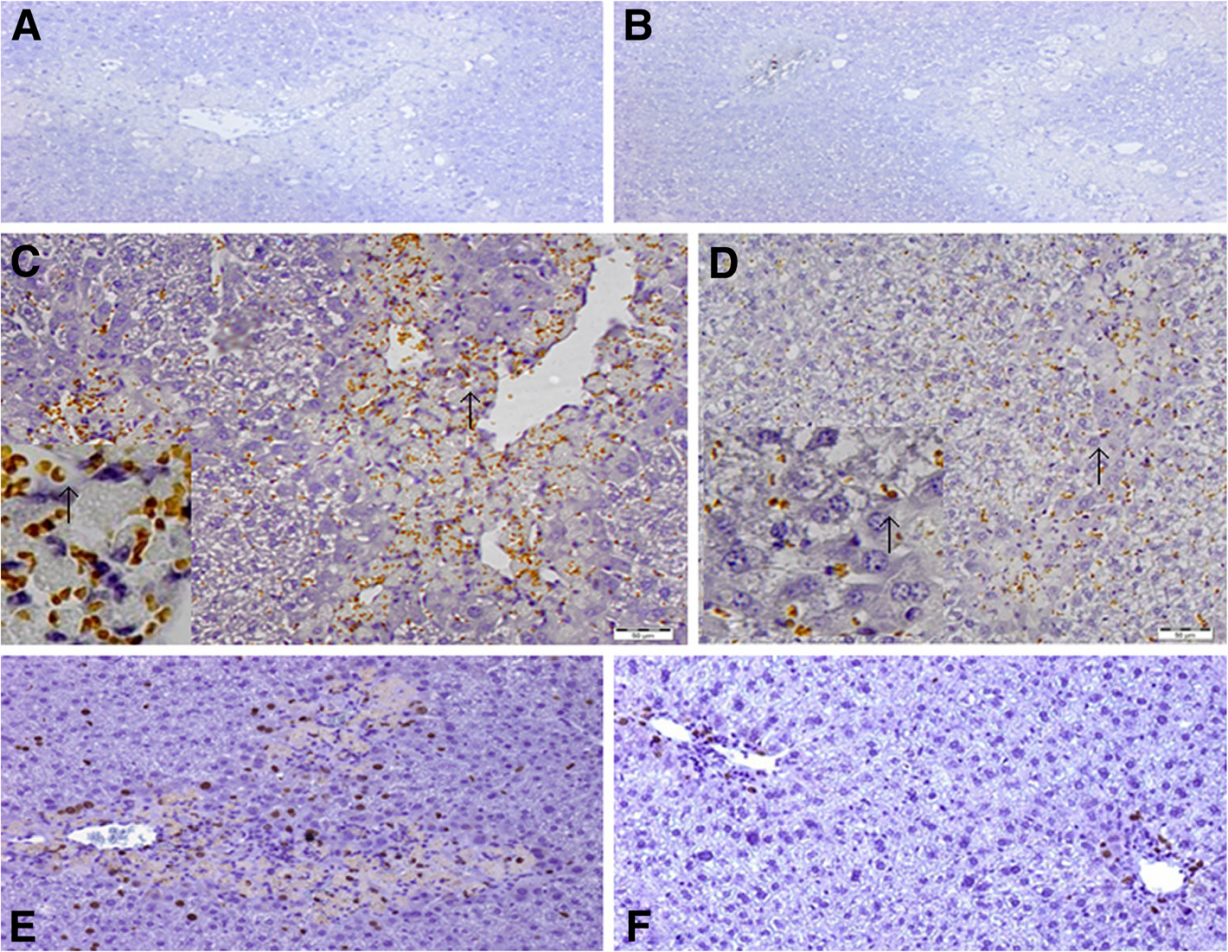
**Effect of treatment with anti-HMGB1 antibody or sham IgG on hepatic proliferation in mice challenged with acetaminophen overdose.** PCNA staining was assessed 24 and 48 hours after acetaminophen administration. Animal model and treatment were the same as described in Figure 1 (n = 6 for each group at 24 h time point, n = 9-10 for each group at 48 h. **A** = sham IgG at 24 h, **B** = anti-HMGB1 at 24 h, **C** = sham IgG at 48 h, **D** = anti-HMGB1 at 48 h, arrows indicate those PCNA-positive nuclei at high power 400×). Figure 6**E** and **F**: Effect of treatment with sham or anti-HMGB1 antibodies on hepatocyte regeneration in acetaminophen injected mice. Animal model and treatment were the same as the 48 h experiment described in Figure 1 except that mice from the 48 h groups were administered 5-bromo-2-deoxyuridine (BrdU, 50-mg/kg i.p. injection at 46 h time point). BrdU staining was assessed at 48 h (n = 6 for each group, **E** = sham IgG at 48 h, **F** = anti-HMGB1 at 48 h, magnification = 200× for **A**, **B**, **C**, **D**, **E** and **F**). A typical picture is shown.

We have shown that in APAP overdose, HMGB1 impairs hepatocyte regeneration and blockade of HMGB1 improves hepatocyte regeneration and liver structure recovery seen in HE staining [22]. Our PCNA staining showed that at 24 h time point, the PCNA staining in the control and sham IgG groups was occasional (1.0 ± 0.1 per high power field) (Figure 6A) and this proliferating tendency was consistent with Javier Vaquero’s report [23], in which BrdU staining was carried out to stain proliferating hepatocytes; however, anti-HMGB1 group consistently showed 14 ± 3 PCNA-positive nuclei per high power field (Figure 6B) (n = 6 for each group), and this number was significantly higher than that in the sham IgG group (p < 0.05). At 48 hours, the total number of PCNA-labeled nuclei was 636 ± 45 per high power field in the sham IgG group (Figure 6C, arrows indicate those PCNA-positive nuclei at higher power 400×). Among the total number of the PCNA-positive nuclei in the sham IgG group, 493 ± 33 were the inflammatory cells that infiltrated in the necrotic area; 72 ± 8 were endothelial cells in the sinusoid; 41 ± 6 were kupffer cells in the sinusoid; 30 ± 3 were other non-parenchymal cells not recognized by nuclear staining. In the anti-HMGB1 group at 48 h, the total number of PCNA-labeled nuclei was 228 ± 14 per high power field (Figure 6D). Among the total number of the PCNA-positive cells in the anti-HMGB1 group, 70 ± 8 were inflammatory cells mainly located in the necrotic area; 63 ± 8 were endothelial cells in the sinusoid; 44 ± 4 were kupffer cells in the sinusoid; 51 ± 6 were other non-parenchymal cells not recognized by nuclear staining. At 48 h, the total number of PCNA-positive nuclei and the number of PCNA-positive inflammatory cells in the sham IgG group were significantly larger than that in the anti-HMGB1 group (*p < 0.05*). Our result was consistent with Javier Vaquero’s report [23], in which larger necrotic area had larger number of cell proliferation. At 48 h, our result showed that the sham IgG group had larger necrotic area (26.6 ± 3.5%) than the anti-HMGB1 group (4.2 ± 0.8%).

### Liver injury and hepatic BrdU staining

24 hrs after APAP injection, anti-HMGB1 therapy did not statistically decrease serum ALT/AST or significantly reduce necrosis as compared with the sham IgG group (n = 6 for each group); however, anti-HMGB1 therapy instead of the sham IgG treatment showed evident hepatocyte regeneration at 24 h (published in reference [22]). 48 hrs after APAP challenge, the sham IgG treated mice showed significantly larger hepatic necrotic area than the anti-HMGB1 treated animals and blockade of HMGB1 significantly decreased serum transaminases (ALT and AST) (published in reference [22]). Similarly, in a new 48 h BrdU experiment, sham IgG treated mice demonstrated 26.6 ± 3.5% necrotic area, which was significantly larger than that in the anti-HMGB1 group (4.2 ± 0.8%); the sham IgG group also had significantly higher serum ALT (480 ± 35 U/L) than that in the anti-HMGB1 group (148 ± 17 U/L, results are means ± SEM, n = 6 for each group).

The hepatocyte proliferation was assessed by BrdU immunohistological staining. At 48 hours, as compared with the control group (the number of BrdU-positive cells in the control group was 1.0 ± 0.1 per high power field), the number of BrdU-labeled nuclei (per high power) was significantly increased in both sham IgG (82 ± 8, Figure 6E) and anti-HMGB1 (18 ± 2, Figure 6F) groups (results are means ± SEM, n = 6 for each group), although to a statistically lesser extent in the anti-HMGB1 treated mice, our result was consistent with Javier Vaquero’s report [23] in which after 48 h, the extent of hepatic BrdU expression, however, depended mainly on the extent of damage, because it was significantly correlated with the area of hepatocyte necrosis for each mouse [23].

## Discussion

HMGB1 is a well-known late inflammatory mediator in sepsis [10] and it can be released readily from necrotic or damaged cells to serve as a signal for inflammation [26]. In APAP overdose, massive necrotic hepatocytes release HMGB1 [9], HMGB1 impairs hepatocyte regeneration and blockade of HMGB1 improves hepatocyte regeneration and liver structure recovery [22]. In this study, our data showed that larger hepatic necrosis was associated with higher serum HMGB1 levels and larger number of BT. At 48 h time point, compared to the sham IgG group, the anti-HMGB1 group had smaller necrotic area, the smaller necrosis likely released less HMGB1 to mediate less bacteria translocation.

The liver not only filters the blood returning from the intestines to remove noxious agents, but also plays an important role in the process of BT [2] by virtue of its many phagocytic cells comprising about 80% to 90% of the reticuloendothelial system function in the body [20]. Additionally, the liver produces acute phase proteins, which act as protease inhibitor or opsonins, the liver actively participates in the body defense mechanisms against infections [27].

Liver regeneration is a vital process for survival after a toxic insult [28,29]. Regeneration ensures the replacement of necrotic cells and the full recovery of organ function. Since hepatocytes are mostly in a quiescent state (G_0_), the regeneration process requires entry into the highly regulated cell cycle [25]. The first step of this process is to prime hepatocytes by cytokines such as TNF-α and IL-6 [25], which makes cells more responsive to growth factors [25]. In current investigation, our PCR data also demonstrated that anti-HMGB1 antibody therapy significantly increased the expression of regeneration-promoting transcripts TNF-α and IL-6 in APAP-treated liver tissue at 48 h time point, suggesting that blockade of HMGB1 likely facilitates activation of TNF-α, IL-6 pathway to restore hepatic structure. Our study also showed that at 24 h, the PCNA staining in the control and sham IgG groups was occasional (1.0 ± 0.1 per high power field), however, anti-HMGB1 group consistently showed 14 ± 3 PCNA-positive nuclei per high power field, and these cells were not hepatocytes, this is not consistent with the results from HE staining in which anti-HMGB1 treatment shows evident hepatocyte regeneration while the sham IgG therapy does not [22], the underlying mechanism is unknown. 48 h after APAP injection, the number of PCNA-labeled nuclei was significantly increased in both the sham IgG and the anti-HMGB1 groups, most of the PCNA-positive cells were inflammatory cells and other non-parencymal cells, suggesting that the hepatic immune function is likely to be compromised to block bacterial translocation through the liver. 48 h after APAP administration, the sham IgG group still had larger hepatic necrosis; in contrast, the anti-HMGB1 treatment showed better recovered liver structure, reduced hepatic bacterial translocation and significantly increased hepatic TNF-α and IL-6 mRNA expression at late phase, suggest that it is possible that liver immune system in the anti-HMGB1 therapy group is recovered better than the sham IgG group. At 48 h, even though the number of PCNA-positive non-parenchymal cells in the sham IgG group was larger than that in the anti-HMGB1 therapy group, the larger number of PCNA positive cells at late phase might indicate larger liver necrotic area (this is consistent with Javier Vaquero’s report [23]) in the sham IgG group than the anti-HMGB1 group, these PCNA-positive cells are in the stage of proliferation and they are immature to function normally; in contrast, at late phase, the smaller number of PCNA-positive cells in the anti-HMGB1 group might indicate less liver injury in the anti-HMGB1 group, and the immune system in this group might be more closer to normal than the sham IgG group, resultantly, anti-HMGB1 treatment decreased BT to the liver because the normal liver structure and normal hepatic immune system are two important factors to process BT in the liver.

24 h after APAP injection, as compared to the normal control group, sham IgG treatment significantly increased TNF-α and IL-6 mRNA levels and the enhanced TNF-α and IL-6 mRNA expression was associated with large necrosis without evident regeneration [22]; in contrast, anti-HMGB1 therapy instead of the sham IgG treatment markedly decreased these two cytokines’ mRNA expression and improved hepatocyte regeneration at early phase [22], this indicates that TNF-α and IL-6 are pro-inflammatory cytokines at early phase and their mRNA expressions are modulated by HMGB1, this result is supported by the following two reports [11,12] in which HMGB1 stimulates macrophages to release TNF-α and IL-6, neutralizing HMGB1 blocks TNF-α release [11,12] and knocking-out HMGB1 receptor reverses IL-6 release [12]. TNF-α and IL-6 are well known as important pro-inflammatory cytokines; however, their roles in APAP overdose are controversial. Knocking out the TNF-α receptor reduces hepatocyte proliferation in APAP toxicity [30], and IL-6 knockout mice are more susceptible to APAP-induced liver injury than the wild type mice [31], suggesting that IL-6 may protect liver from injury. In current study at 48 h, anti-HMGB1 treatment instead of the sham IgG therapy markedly enhanced hepatic TNF-α and IL-6 mRNA levels, the increase in TNF-α and IL-6 at later phase could be due to the fact that macrophages (the classically activated M1 and alternatively activated M2 populations) are proliferating and/or migrating into the liver [32,33], and the M1 subset macrophages might play hepatotoxic role at early stage of ALF, while the M2 subset macrophages likely play hepatoprotective role at late phase of ALF [32]; it is also possible that hepatocytes could also produce these cytokines and their regeneration can be responsible for this increase. The increased TNF-α and IL-6 expression at late phase was associated with better recovered hepatic structure, these results indicate that inflammation might play different roles at different phases in APAP overdose: inflammation likely contributes to liver injury at early phase but also likely improves hepatocyte regeneration and liver recovery at late phase. This notion is supported by the following APAP overdose studies [21,24,34] in which Ringer’s lactate, the pro-inflammatory solution, increases serum TNF-α and improves liver recovery at late phase while the anti-inflammatory agent ethyl pyruvate (EP) reduces liver injury at early phase but impairs hepatic regeneration at late phase; kupffer cells (KC) are important source of TNF-α and IL-6 and play important roles in inflammation, and KC depletion confers protection at early time points after APAP treatment but can lead to more severe injury at later time point [34].

Gut mucosal barrier plays an important role in BT; the intact gut mucosal barrier is essential to prevent intestinal bacterial translocation. When the integrity of intestinal mucosa is impaired, it would be easier for gut luminal bacteria to adhere to the injured mucosa to facilitate BT. In current study, as compared to the control group, the gut mucosal permeability was significantly increased in the APAP challenged mice at both 24 h and 48 h, the mucosal permeability in the sham IgG treatment groups (both 24 h and 48 h) was not significantly higher than that in the anti-HMMGB1 therapy groups; however, as compared to the sham IgG treatment groups, HMGB1 neutralizing antibody treatment blocked about 85% of BT at both early and late time points, the results indicate that BT is likely a HMGB1-mediated (at least partly) active procedure, in which, except the impaired mucosal integrity, HMGB1 is also needed and it is unlikely that gut luminal bacteria passively translocate via the damaged mucosal paracellular tight junction, further investigation is needed to clarify the underlying mechanism.

Gut permeability and bacterial translocation are positively correlated in hemorrhagic shock model [17] and endotoxemic model [14,35]; however, emerging evidence shows that gut permeability and bacterial translocation could be disassociated in the following two animal models [36,37]: in an alcoholic liver injury model [36], ethyl pyruvate (EP), an potent anti-inflammatory agent, which has been reported to be able to reduce gut bacterial translocation but it does not decrease gut permeability; in a severe acute pancreatitis model [37], EP inhibits gut bacterial translocation but does not reduce ileal mucosal permeability. The underlying mechanism is still not clear; more studies are needed to clarify this issue.

Collectively, our results support the notion that HMGB1 is an important factor, which links the APAP hepatotoxicity and gut bacterial translocation; once the enteric bacteria enter the systemic circulation, it likely triggers the systemic inflammatory response (SIRS) and leads to sepsis, MOF [38] and death in APAP hepatotoxicity.

## Conclusion

HMGB1 neutralization is associated with bacterial translocation during acetaminophen hepatotoxicity; blockade of HMGB1 may present a novel therapy to prevent multiple organ failure from gut bacterial translocation in APAP overdose.

## Abbreviations

APAP: Aacetaminophen; EP: Ethyl pyruvate; I.P.: Intraperitoneal; HMGB1: High mobility group B1; BT: Bacterial translocation; PCNA: Proliferating cell nuclear antigen; ALF: Acute liver failure; MOF: Multiple organ failure.

## Competing interests

The authors declare that they have no competing interests.

## Authors’ contributions

RKY designed the study. MLK performed immunohistostaining. JT wrote manuscript. All authors participated in the animal handling and procedures. All authors read and approved the final manuscript.

## Pre-publication history

The pre-publication history for this paper can be accessed here:

http://www.biomedcentral.com/1471-230X/14/66/prepub
